# Structure-Function Model for Kissing Loop Interactions That Initiate Dimerization of Ty1 RNA

**DOI:** 10.3390/v9050093

**Published:** 2017-04-26

**Authors:** Eric R. Gamache, Jung H. Doh, Justin Ritz, Alain Laederach, Stanislav Bellaousov, David H. Mathews, M. Joan Curcio

**Affiliations:** 1 Laboratory of Molecular Genetics, Wadsworth Center, New York State Department of Health, Albany, NY 12201, USA; egamache@iu.edu (E.R.G.); junghdoh@gmail.com (J.H.D.); 2 Department of Biology, University of North Carolina, Chapel Hill, NC 27599, USA; justin.ritz1@gmail.com (J.R.); alain@unc.edu (A.L.); 3 Department of Biochemistry and Biophysics and Center for RNA Biology, University of Rochester Medical Center, Rochester, NY 14642, USA; stasuofr@gmail.com (S.B.); David_Mathews@URMC.Rochester.edu (D.H.M.); 4 Department of Biomedical Sciences, University at Albany-SUNY, Albany, NY 12201, USA

**Keywords:** long terminal repeat-retrotransposon, Ty1, *Saccharomyces cerevisiae*, RNA secondary structure, RNA packaging, RNA kissing complex, pseudoknot, kissing loop, SHAPE analysis

## Abstract

The genomic RNA of the retrotransposon Ty1 is packaged as a dimer into virus-like particles. The 5′ terminus of Ty1 RNA harbors *cis*-acting sequences required for translation initiation, packaging and initiation of reverse transcription (TIPIRT). To identify RNA motifs involved in dimerization and packaging, a structural model of the TIPIRT domain in vitro was developed from single-nucleotide resolution RNA structural data. In general agreement with previous models, the first 326 nucleotides of Ty1 RNA form a pseudoknot with a 7-bp stem (S1), a 1-nucleotide interhelical loop and an 8-bp stem (S2) that delineate two long, structured loops. Nucleotide substitutions that disrupt either pseudoknot stem greatly reduced helper-Ty1-mediated retrotransposition of a mini-Ty1, but only mutations in S2 destabilized mini-Ty1 RNA in *cis* and helper-Ty1 RNA in trans. Nested in different loops of the pseudoknot are two hairpins with complementary 7-nucleotide motifs at their apices. Nucleotide substitutions in either motif also reduced retrotransposition and destabilized mini- and helper-Ty1 RNA. Compensatory mutations that restore base-pairing in the S2 stem or between the hairpins rescued retrotransposition and RNA stability in *cis* and trans. These data inform a model whereby a Ty1 RNA kissing complex with two intermolecular kissing-loop interactions initiates dimerization and packaging.

## 1. Introduction

Long terminal repeat (LTR)-retrotransposons and related families of endogenous retroviruses are mobile genetic elements that are widespread in eukaryotic genomes. These elements encode the enzymatic machinery to reverse transcribe RNA and integrate the resulting cDNA into the host genome. They mobilize their own RNA, that of non-autonomous mobile elements, and, more rarely, “hitchhiker” transcripts including coding and non-coding RNAs. The genomic incorporation of cDNA derived from cellular RNAs results in the duplication or replacement of cellular genes and the formation of novel chimeric genes and regulatory non-coding genes, insertional mutations and chromosomal rearrangements [1,2,3,4]. In *Saccharomyces cerevisiae*, for example, it has been argued that most protein coding genes have been replaced with cDNA copies lacking introns through the activity of retrotransposons [5,6,7]. In addition, chimeric cDNAs are incorporated at telomere ends in the absence of telomerase, leading to gross chromosomal rearrangements [8]. Because of the mutagenic and regulatory potential of cDNAs derived from cellular transcripts, the factors that govern the specificity of RNA selection for reverse transcription are of great interest, yet little is known about the principles that govern recognition of RNAs for packaging into virus-like particles (VLPs), the site of reverse transcription. This question is addressed here by investigating the determinants of Ty1 RNA packaging. Ty1 is the most active LTR-retrotransposon family in *S. cerevisiae* [9]. The positive-strand genomic Ty1 RNA initiates in the 5′ LTR and terminates in the 3′ LTR. Ty1 RNA is translated into p49-Gag and p199-Gag-Pol precursor proteins. These proteins assemble into an immature VLP, with p49-Gag binding to Ty1 RNA as a dimer to encapsidate the RNA genome [10]. Inside the VLP, p49-Gag is processed to form p45-Gag, resulting in VLP maturation, which in turn results in stabilization of the Ty1 RNA dimer. The p199-Gag-Pol precursor is processed into p45-Gag, protease (PR), integrase (IN) and reverse transcriptase (RT). Ty1 RNA functions as a template for synthesis of cDNA that is transported to the nucleus and integrated into the genome.

**Figure 1:**
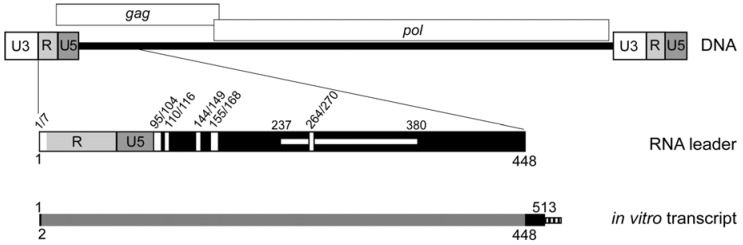
Schematic of the Ty1 element DNA, the Ty1 RNA 5′ TIPIRT domain and in vitro transcripts analyzed by SHAPE chemistry. Ty1 retrotransposon DNA consists of two 334 bp long terminal repeats (LTRs; represented by tripartite rectangles) composed of U3 (unique to the 3′ end of the RNA), R (repeated at the 5’ and 3′ ends of the RNA) and U5 (unique to the 5’ end of Ty1 RNA). LTRs flank a central coding region (black bar). The *GAG* and *POL* ORFs are denoted by rectangles above the element. Below the DNA, the 5′ leader of Ty1 RNA from nucleotide 1 (the beginning of “R”) to nucleotide 448 (in the *GAG* ORF), which includes the TIPIRT domain (nucleotides 1–380), is represented below the Ty1 element DNA. Vertical white rectangles denote sequences that are essential for initiation of reverse transcription (1/7 and 264/270 pseudoknot S1 stem; 95/104-PBS; 110/116-Box 0; 144/149-Box 1; 155/168-CYC5, including Box 2.1). The horizontal white rectangle spanning nucleotide 237–380 denotes a region required for Ty1 RNA packaging. The schematic at the bottom represents the in vitro transcript (nucleotides 1–513, plus an FTL tag indicated by the striped box) that was analyzed by SHAPE. Grey shading (nucleotide 2–448), region for which SHAPE reactivities were obtained.

A domain of Ty1 RNA consisting of the 53-nucleotide 5′ UTR and 327 nucleotides of the *GAG* coding region are required in *cis* for translation initiation, packaging and the initiation of reverse transcription (TIPIRT domain; Figure 1) [11]. Mutational analysis has identified several RNA motifs within the TIPIRT domain that play a role in reverse transcription. These regions include the primer-binding site (PBS; nucleotides 95–104), which is complementary to the 3′ end of tRNA_i_^Met^. The tRNA_i_^Met^ is selectively packaged into Ty1 VLPs and serves as the primer for initiation of reverse transcription [12,13]. Three adjacent 6- or 7-nucleotide regions of TIPIRT, known as Box 0 (nucleotides 110–116), Box 1 (nucleotides 144–149) and Box 2.1 (nucleotides 162–168) [14,15], are complementary to sequences within the T or D hairpins of tRNA_i_^Met^. Analyses of mutations in both Ty1 RNA and tRNA_i_^Met^ have established a role for an extended interaction between tRNA_i_^Met^ and the PBS, Box 0 and Box 1 regions of Ty1 RNA in the initiation of reverse transcription [15,16]. Overlapping Box 2.1 is a 14-nucleotide motif known as CYC5 (nucleotides 155–168), which is perfectly complementary to a sequence in the 3′ UTR known as CYC3. CYC5:CYC3 complementarity promotes efficient reverse transcription in vitro and retrotransposition in vivo [17,18]. In addition, intramolecular pairing of nucleotides 1–7 to nucleotides 264–270 promotes efficient reverse transcription [19,20].

A secondary structure model of the 5′ terminus of Ty1 RNA within VLPs was derived from SHAPE (selective hydroxyl-acylation analyzed by primer extension) data [21]. In this model, nucleotides 1–325 form a long-range pseudoknot in virio. The pseudoknot core consists of two 7 bp stems with a 1-nucleotide interhelical connector, and long structured loops that bridge the stems. The model supports many aspects of earlier structural models that were based on secondary structure prediction and mutational analyses [16,19], including pairing of the tRNA_i_^Met^ to the PBS, Box 0 and Box 1 regions of TIPIRT and circularization of Ty1 RNA via the CYC5:CYC3 interaction. Moreover, the functionally defined pairing of nucleotides 1–7 to nucleotides 264–270 forms the S1 stem of the pseudoknot. All of the RNA motifs that are known to be required for reverse transcription are in S1 or its multibranched loop (L1), suggesting that this domain may be functionally as well as structurally distinct from S2 and its loop (L3). Using nucleotide substitutions and compensatory mutations, it was shown that the S2 stem is required for retrotransposition, but, an S2 stem-destabilizing mutation, U260C, had no effect on reverse transcription [20].

In contrast with *cis*-acting sequences required for reverse transcription, Ty1 RNA sequences that are necessary for dimerization and packaging within VLPs have not been precisely defined [22]. An internally deleted mini-Ty1 RNA containing the 380-nucleotide TIPIRT domain and 357 nucleotides of the 3′ terminus of Ty1 RNA including the 3′ polypurine tract and 3′ LTR, was shown to be sufficient for retrotransposition when *GAG* and *POL* proteins were expressed in *trans* from a helper-Ty1 element [11]. Deletion of nucleotides 237–380 abolished retrotransposition and co-purification of mini-Ty1 RNA with VLPs, suggesting that *cis-*acting sequences required for Ty1 RNA packaging reside in this domain. This region includes one strand of the S1 stem as well as the S2 stem and its structured loop [21]. However, mutations that destabilize S1 pairing, or the U260C mutation in the S2 stem did not diminish Ty1 RNA packaging [20].

RNA elements required for encapsidation of retroviral RNA within virions, known as ψ (psi) sites, are at least 100 nucleotides long, contain multiple stem-loop structures and are in the 5′ UTR, sometimes extending into *GAG*. RNA elements that facilitate dimerization are located near those that promote RNA encapsidation, and dimerization and packaging are tightly coupled processes, both facilitated by the nucleocapsid activity of Gag [23,24]. Prior to recruitment into assembling virions, dimerization of retroviral genomes is initiated by an intermolecular “kissing loop” interaction between single-stranded loop sequences of stem-loops in the RNA. Subsequently the interaction extends into palindromic sequences in the stems to form stable dimers. Purzycka et al. [21] identified three palindromic sequences (PAL1–PAL3) in the 5′ terminus of Ty1 RNA that were less reactive in virio than ex virio. Based on analogy with retroviral dimerization sites, the authors proposed that PAL sequences are sites where the nucleic acid chaperone activity of Gag could promote a transition from intramolecular pairing to intermolecular pairing [25]. However, potential kissing loop sequences that initiate dimerization of Ty1 RNA have not been identified.

In this work, we present a SHAPE-directed structural model of the 5′ TIPIRT domain of Ty1 RNA in vitro. The model corroborates previously proposed models of the 5′ terminus of Ty1 RNA in virio and in vitro [20,21]. We overlay nucleotide conservation of *Saccharomyces* Ty1 and Ty2 element sequences onto the secondary structure model to identify conserved secondary structures with potential roles in packaging. The biological significance of structural elements was investigated by introducing mutations into mini-Ty1 RNA and measuring helper-Ty1-mediated retrotransposition in vivo. We confirmed the requirement for both stems of the pseuodoknot in retrotransposition [20], and found that separating the stems by four nucleotides has no effect on retrotransposition, raising the possibility that the stems play roles in non-overlapping functions. In addition, complementary 7-nucleotide motifs at the apices of two stem-loops, SL1a and SL3a, were shown to be required for efficient retrotransposition. Unlike S1 stem mutations [20], nucleotides substitutions in the S2 stem subject Ty1 RNA to rapid degradation in *cis* and in *trans.* Also, SL1a and SL3a loop substitutions result in slow and fast degradation, respectively, in *cis* and in *trans*. Trans-complementation of the helper-Ty1 RNA instability by compensatory mutations in the mini-Ty1 RNA SL1a and SL3a apical motifs suggests that these motifs form intermolecular duplexes. Based on these data, we propose that intermolecular pairing between the apical motifs of these stem-loops, one of which has been implicated in dimerization [21], and the other of which may be dependent on the S2 stem for its stability, forms a Ty1 RNA kissing complex that initiates dimerization of Ty1 RNA for packaging into VLPs.

## 2. Materials and Methods

### 2.1. In Vitro Transcription and RNA Purification

The DNA template for in vitro transcription was generated by PCR with primers PJ502 (5′-CCTAATACGACTCACTATAGGGGAGGAGAACTTCTAGTATATTCTG-3′) and PJ745 (5’-ATGAGCTCCCAGATTCGTCAGAATTATCAGTAAATGTATTACCTGACTCAGG-3′) and plasmid pGTy1*his3AI-[∆1*] [26] as a template. The reaction yielded a DNA fragment with the T7 promoter, 513 bp corresponding to nucleotide 1–513 of Ty1-H3 RNA and 27 bp complementary to the Cy5-FTL primer. The PCR product was gel-purified using a Gel Extraction kit (Qiagen, Germantown, MD, USA). In vitro transcription reactions were performed using ~150 ng of the purified DNA template in a 20 μL MEGAscript T7 transcription kit (Invitrogen, Carlsbad, CA, USA) reaction, which was incubated at 37 °C for 4 h. The RNA was purified using the MEGAclear kit (Invitrogen). RNA was stored at −80 °C.

### 2.2. Selective 2′-Hydroxyl Acylation Analyzed by Primer Extension

A 10 picomole sample of the in vitro transcribed RNA was brought to a total volume of 12 μL by addition of 0.5× TE. The RNA was heated at 95 °C for 2 min and cooled on ice for 2 min. Following addition of 6 μL 3.3× RNA Folding Buffer (1×: 100 mM HEPES [pH 8.0], 20 mM MgCl_2_, 100 mM KCL), the reaction was split into two samples of equal volume. RNA was renatured by incubation at 37 °C for 20 min. To one sample, 1 μL *N*-methylisotoic anhydride (NMIA) in DMSO was added, and to the other, 1 μL DMSO was added (control). Reactions were incubated at 37 °C for 45 min. The NMIA-modified RNA and control RNA samples were ethanol-precipitated by adding 90 μL H_2_O, NaCl to 44 mM, glycogen to 44 μg/μL and EDTA to 44 μM. After adding 3.5× volumes of ethanol, the RNA was precipitated at −80 °C for 30 min. The RNA was pelleted at 4 °C, and washed with 70% ethanol. Pellet was dried in a Savant SpeedVac concentrator and resuspended in 10 µL of 0.5× TE buffer (1×: 10 mM Tris-HCl [pH 8], 1 mM EDTA). The Cy5-FTL primer (5’-ATAATTCTGACGAATCTGGGAGCTCAT-3’) was annealed to the 3’ end of each RNA at 65 °C for 15 min, and then 35 °C for 15 min. RNA was reverse transcribed by first adding Superscript III First-Strand Synthesis buffer (Invitrogen), 5 mM DTT, 40U of RNAseOUT (Invitrogen), and 500 μM dNTPs to the reaction, followed by incubation at 52 °C for 1 min. Superscript III Reverse Transcriptase (200 units; Invitrogen) was added, and the reaction was incubated at 52 °C for 15 min. RNA was hydrolyzed by addition of NaOH to 180 mM, followed by heating to 95 °C for 5 min. Reactions were neutralized by addition of an amount of HCl equivalent to the NaOH added. The remaining nucleic acid was precipitated using sodium acetate at a final concentration of 75 mM, MgCl_2_ at 25 mM, and 3.3× volumes of ethanol followed by cold centrifugation. The resulting pellet was washed with 70% ethanol and dried in a Savant SpeedVac concentrator. The pellet was resuspended in 40 μL Sample Loading Solution (Beckman Coulter, Indianapolis, IN, USA), and 1.1 μL of the 600-bp Beckman Coulter sequencing ladder was added. Sequencing ladder reactions were performed in the same way as the control reaction above, with addition of 2 μL of one 5 mM dideoxyNTP (ddNTP). Two different ddNTP reactions were run for each sample, using a different ddNTP for each. Primer extension products were resolved by capillary electrophoresis using a Beckman Coulter CEQ8000 Genetic Analysis System.

Experimental datasets from three technical replicates were individually corrected for signal variation, and peak intensities were integrated using MatLab (MathWorks, Inc., Natick, MA, USA) and ShapeFinder [27]. Reactivities were normalized by dividing the peak intensities by the average of the 10% most reactive peaks excluding outliers, which were determined by boxplot analysis as those peaks showing reactivity greater than 1.5× the interquartile range. The standard deviation (SD) of the normalized reactivities of each nucleotide position was calculated across datasets, and reactivities with SD > 0.7 were also excluded. Normalized reactivities of overlapping nucleotides from the three RNA species were averaged to obtain a composite dataset spanning nucleotides 1 to 615 of the Ty1 RNA. Composite reactivity data was used to determine a pseudo-free energy change restraint added to the nearest-neighbor thermodynamic parameters [28,29]. Structure prediction was performed using ShapeKnots [30]. Collapsed diagrams were generated using XRNA (http://RNA.ucsc.edu/RNAcenter/xRNA/xRNA.html). Diagrams were edited using Adobe Illustrator. The raw SHAPE data is available in SNRNASM format as supplemental data in this manuscript (Table S1) [31].

### 2.3. Conservation of Sequences in the Ty1 RNA 5' Terminus

Clustal X [32] was used to align sequences corresponding to nucleotides 1 to 615 of the transcript of 31 genomic Ty1 elements and 15 Ty2 elements in *S. cerevisiae* strain S288C (www.yeastgenome.org), 35 Ty1 and 17 Ty2 sequences from other strains of *S. cerevisiae* (www.ncbi.nlm.nih.gov/genome?term=txid4932[orgn]), and four Ty1-like elements from other *Saccharomyces* species, including one element from *Saccharomyces weihenstephen* (accession number gb|ABPO01001678.1|); one element from *Saccharomyces* *mikatae* (gb|AACH01000084.1|); one element from *Saccharomyces paradoxus* (gb|AABY01000078.1|); and one element from *Saccharomyces kluyveri* (gb|AF492702.1|). Each nucleotide position was assigned to one of three categories based on whether the nucleotide was conserved in all 102 Ty elements and if not, whether the nucleotide was conserved in the 66 Ty1 elements of *S. cerevisiae*.

### 2.4. Plasmids

The helper-Ty1 plasmid, pEIB, was a kind gift of Leslie Derr and Jeffrey Strathern. It is a 2 µ-based, *TRP1*-marked plasmid harboring the *GAL1* promoter fused to nucleotides 241-5561 of Ty1-H3 DNA [33]. This region of Ty1-H3 includes the R and U5 regions of the 5’ LTR and *GAG* and *POL* ORFs, but lacks the plus-strand polypurine tract (PPT1) and the 3’ LTR. The Ty1-H3 sequence in pEIB also harbors mutations (T335C, T338A, A339T, G340C, C341A, C344A, T347C) that disrupt annealing of the tRNA_i_^Met^ primer but preserve the amino-acid sequence of *GAG*.

The mini-Ty1*his3AI* plasmid, pJC994, is a 2 µ-based, *URA3*-marked plasmid that was constructed by deleting the HpaI-SnaBI fragment of pGTy1*his3AI*-*[∆1]* (nucleotides 818–5463 of Ty1-H3 DNA) [26]. Mutations were introduced into plasmid pJC994 using the QuikChange Lightning Site-Directed Mutagenesis kit (Agilent Technologies, Santa Clara, CA, USA) and the standard protocol. Plasmid DNA was purified and Ty1 sequences were confirmed by DNA sequencing. The sequence of primers used for site-directed mutagenesis is available upon request.

Plasmid p*GAL1:GAG_NT_:GFP* is a CEN-based *LEU2*-marked plasmid consisting of vector pRS415 carrying an ApaI-EagI fragment containing the *GAL1* promoter, the 575 bp XhoI-HpaI fragment of Ty1-H3 (nucleotides 241–815), a 7-nucleotide linker including a *Bam*HI site, and the *GFP(S65T)* ORF and *ADH1* terminator from plasmid pFA6-*GFP(S65T)-HIS3MX* [34]. Mutations in Ty1-H3 sequence were introduced into p*GAL1:GAG_NT_:GFP* by PCR-amplification of Ty1 sequences from derivatives of pJC994 containing various mutations in mini-Ty1, digestion with *Xho*I-*Bam*H1, and substitution of the resulting fragment for the XhoI-BamHI fragment of p*GAL1:GAG_NT_:GFP*.

### 2.5. Quantitative Transposition Frequency Assay

Plasmids pEIB and pJC994 or its mutagenized derivatives were co-transformed into strain JC5839 (*MAT*a *his3∆1 ura3∆0 leu2∆0 met15∆0 trp1::hisG spt3∆::kanMX*), a derivative of strain BY4741 [35]. Single colony isolates of each strain grown in SC-Ura-Trp 2% glucose broth at 30 °C for 2 days were pelleted and resuspended in 5 volumes of SC-Ura-Trp 2% galactose, 2% raffinose broth. Each culture was divided into seven 1-mL cultures, which were grown at 20 °C. After 48 h, 1 mL of YEPD broth was added to each culture, which was incubated at 20 °C for 18 h. A 1 µL aliquot of each culture was removed, and dilutions were plated onto YEPD agar to determine the total number of colony forming units. Aliquots of the remaining culture were plated onto SC-His 2% glucose agar. The retrotransposition frequency for each culture is the number of His^+^ prototrophs divided by the total number of colony forming units in the same volume of culture. The median retrotransposition frequency among the seven biological replicates was determined, and the 95% confidence interval was calculated.

### 2.6. Northern Analysis

Single colony isolates of each strain were grown in SC-Ura-Trp 2% glucose broth at 30 °C. Cells were pelleted, washed in water, and resuspended in SC-Ura-Trp 2% galactose 2% raffinose broth at an OD_600_ of 0.05 and grown for 20–24 h at 20 °C to an OD_600_ of 0.4. Cells were pelleted and washed in water, and cell pellets were frozen at −80 °C. RNA was extracted from cell pellets thawed on ice for 30 min using the MasterPure (Epicentre, Madison, WI, USA) kit. A 10 µg sample of total RNA and an equal volume of Ambion NorthernMax glyoxal loading buffer (Thermo Fisher Scientific, MA, USA) was incubated for 30 min at 50 °C. Samples were fractionated on a 1% agarose gel in 10 mM NaPO_4_, pH 6.5 at 100 V for 2.5 h. RNA was transferred to a Hybond-XL (GE Healthcare, Troy, NY, USA) membrane using alkaline transfer conditions for 3 h, and then crosslinked to the membrane using a Spectrolinker (Spectronics Corporation, Westbury, NY, USA) set to “optimal”. In vitro transcribed RNA probes were synthesized using SP6 or T7 polymerase in conjunction with ^32^P-rCTP. Antisense Ty1 RNA (nucleotides 815–2173) transcribed from plasmid pGEM-TyA1 [36] was used to specifically detect helper-Ty1 RNA, sense-strand *HIS3* transcript from plasmid pGEM-HIS3 [36] detected mini-Ty1*his3AI* RNA, and an antisense 18S rRNA transcript from plasmid pBDG512 [37] detected 18S rRNA. Probes were incubated sequentially in NorthernMax hybridization buffer (Ambion). After washing, blots were exposed to phosphor screens and scanned using a Typhoon phosphorimager (GE Healthcare). Images were quantitated using ImageQuant software (GE Healthcare) by normalizing to the 18S rRNA signal. Blots were stripped in boiling 0.1% SDS, rinsed and stored in 5× SSC before reprobing.

### 2.7. GFP Activity

Plasmid p*GAL1:GAG_NT_:GFP*, derivatives bearing nucleotide substitutions and vector pRS415 were transformed into the *spt3∆::kanMX* derivative of strain BY4741 [35]. Two transformants of each plasmid were grown in SC-Leu 2% glucose overnight at 30 °C. Cells were spun down, and pellets resuspended in an equal volume of SC-Leu 2% raffinose 2% sucrose. A 1:20 dilution in SC-Leu 2% raffinose 2% sucrose broth was grown overnight at 20 °C. Cultures were diluted to an OD_600_ of 0.2 and grown for 3 h at 20 °C. Galactose (2% final) was added and cultures were incubated for 2.5 h at 20 °C. A 1 mL aliquot of each culture was spun down at 1000× *g* for 10 min. The medium was aspirated and cell pellets were resuspended in 500 µL sterile water. The geometric mean of the GFP activity in 10,000 cells was quantified by flow cytometry using a FACSCalibur (Becton, Dickinson and Company, Franklin Lakes, NJ, USA), and the average of the geometric mean of GFP activity in the two biological replicates of each strain was determined.

## 3. Results

### 3.1. Secondary Structure Model of Ty1 RNA TIPIRT Domain

The goal of this study was to identify RNA secondary structures and motifs within the Ty1 TIPIRT domain that are involved in RNA dimerization and packaging into VLPs. To begin, a secondary structure model of the Ty1 RNA leader sequences was developed using average SHAPE reactivities and the ShapeKnots algorithm [30]. SHAPE analysis involves treating a folded RNA with an electrophilic agent that forms 2′-*O*-ester adducts with reactive nucleotides in RNA. The SHAPE reactivity of each nucleotide is inversely correlated to the contribution of that nucleotide to base-pairing or tertiary interactions. Adduct formation on each nucleotide is measured as the degree of impediment to primer extension by reverse transcriptase. The ShapeKnots algorithm [30] combines a pseudoknot discovery algorithm with one that reconciles experimental SHAPE reactivities with traditional free energy rules to obtain a structure that is maximally compatible with the experimental data.

An in vitro transcript corresponding to nucleotides 1–513 of Ty1-H3 RNA, which encompasses the Ty1 TIPIRT domain, plus a 27-nucleotide tag was subject to SHAPE analysis (Figure 1). The transcript was folded in 100 mM KCl and 6.7 mM MgCl_2_ and then treated with *N*-methylisotoic anhydride (NMIA), which forms 2′-*O*-ester adducts with reactive nucleotides. The reaction was performed under conditions that promote the formation of a single adduct per RNA molecule. The reactivity of individual nucleotides was determined by reverse transcriptase-mediated primer extension analysis of the transcript that was treated with NMIA or, as a control, untreated. Extension reactions were performed using a fluorescently labeled primer hybridized to the 27-nucleotide tag at the 3′ end of the transcript. The products of primer extension reactions were resolved by capillary electrophoresis. Nucleotides modified by 2′-*O*-adducts were detected as stops to primer extension, resulting in a peak. The reactivity of each nucleotide was determined by integrating individual peaks from NMIA-treated samples. Three independent repetitions were performed and the average SHAPE reactivity at each nucleotide was determined. The average SHAPE reactivities were used to restrain computational predictions of secondary structure models by the ShapeKnots algorithm.

**Figure 2:**
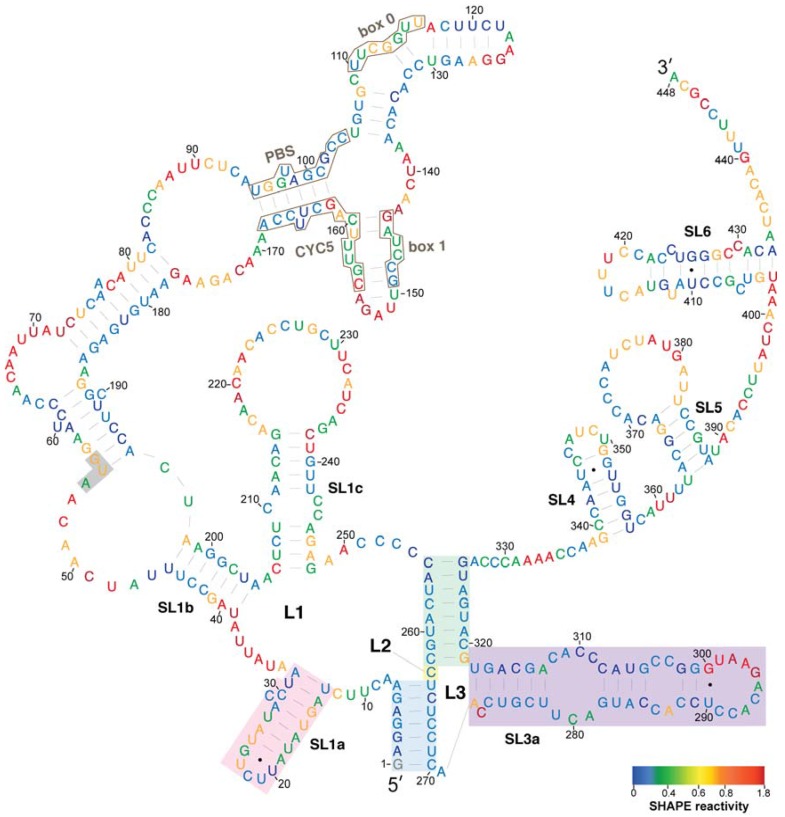
SHAPE reactivities and secondary structure model of the 5′ leader of Ty1 RNA. Nucleotides are colored according to their SHAPE reactivities, which are indicated on the color bar at the bottom left. Regions of low reactivity have a high probability of being constrained within secondary or tertiary structure. The AUG nucleotides shaded in grey comprise the start codon of *GAG*. The pseudoknot core contains stem S1 (blue shading), loop L2 (nucleotide 263, yellow shading), and stem S2 (green shading). Pseudoknot loops L1 (nucleotides 9–254) and L3 (nucleotides 271–318) are not shaded. The SL1a hairpin (pink shading) and SL3a hairpin (purple shading) are indicated.

A model of the secondary structure of the Ty1 RNA TIPIRT domain annotated by the average SHAPE reactivity of each nucleotide position is shown in Figure 2. A prominent feature of the model is a pseudoknot formed by long-range interactions of sequences spanning the first 326 nucleotides of Ty1 RNA, which is within the functionally defined 380-nucleotide TIPIRT domain. This pseudoknot is similar to those predicted previously in the 5′ terminus of in vitro transcribed Ty1 RNA and in Ty1 RNA isolated from VLPs, although earlier modeling did not make use of a pseudoknot discovery algorithm [20,21]. The pseudoknot core consists of the 7-bp S1 pairing (Figure 2, blue shading) and the 8-bp S2 pairing (Figure 2, green shading) connected by a 1-nucleotide interhelical loop (L2) (Figure 2, yellow shading). The S1 stem of the pseudoknot, formed by pairing of the seven 5'-terminal nucleotides of Ty1 RNA to nucleotides 264–270, has an established function during retrotransposition [19,20]. Nucleotides 255–262 interact with nucleotides 319–326 of Ty1 RNA to form the S2 pairing of the pseudoknot (Figure 2, green shading). The S2 stem contains an additional base-pair (C255–G326) that was not predicted in earlier models [20,21].

All but one of the nucleotides within the pseudoknot core had low reactivity with NMIA, including the unpaired L2 nucleotide, suggesting that the pseudoknot is a thermodynamically stable tertiary interaction within the Ty1 RNA. This conclusion is supported by the fact that other RNA structure prediction algorithms that do not employ SHAPE data, such as pknotsRG and IPknot [38,39], also predict a pseudoknot with identical S1 and S2 stems and L2 nucleotide in the 5′ leader of Ty1 RNA (Figures S1 and S2).

The multibranched L1 loop (8/254) of the pseudoknot, formed by stem S1, contains three nested stem-loops (SL1a-SL1c). The first stem-loop (13/32; SL1a) has a single bulged nucleotide and short loop (Figure 2, pink shading). PAL1 and PAL2 sequences, which were proposed to interact intermolecularly in the dimeric RNA of VLPs [25], are contained in the SL1a hairpin. The second stem-loop (39/204; SL1b) is an extended domain containing two nested stem-loops. SL1b contains the sequences that pair with tRNA_i_^Met^ and with 3′ terminal sequences of Ty1 RNA (Figure 2, black outlines) in the model of Ty1 gRNA in virio [21]. The third (206/248; SL1c) is a stem-loop with a bulge loop and an internal loop. L1 sequences include the entire 5′ UTR (1/53) of Ty1 RNA and the AUG codon of *GAG* (Figure 2, highlighted in grey).

The L3 loop of the pseudoknot (271/318) is formed by the S2 pairing and composed almost entirely of the low reactivity SL3a stem-loop (272/318) (Figure 2, purple shading), which has two small internal loops. S2 and L3 are within a region of Ty1 RNA that is necessary for packaging into VLPs (238/380) [11]. Beyond the pseudoknot, the 3′ terminal region of the Ty1 in vitro transcript harbors three stem-loops, SL4, SL5 and SL6. SL6 contains PAL3 (423/428), a putative site of Ty1 RNA dimerization in VLPs [21].

Regions of the structural model that differ from previous SHAPE analysis-derived structural models of Ty1 RNA in virio [21] and the 5′ terminus of Ty1 RNA in vitro [20] include: (a) the presence of the 255C-326G base-pair in the S2 pseudoknot stem, as noted above; (b) extension of the SL1a stem by two base-pairs by inclusion of a 1-nucleotide bulge in our model; (c) the presence of a large loop at the apex of stem-loop SL1c in our model, compared to a bulge-stem-loop structure at the apex of SL1c in previous models; (d) extension of the SL3a stem-loop by two base-pairs by inclusion of a 1-nucleotide bulge in our model; and (e) the presence of SL4, which is not present in previous models. As expected, no evidence of interactions seen in virio between motifs in SL1b and tRNA_i_^Met^ or between CYC5 and CYC3 was observed because neither tRNA_i_^Met^ nor CYC3 are present in our system.

**Figure 3:**
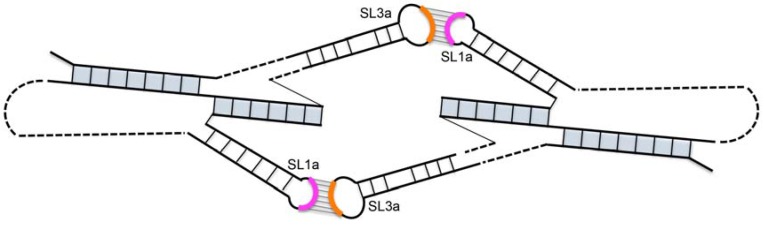
Model of a symmetrical Ty1 RNA kissing complex containing two Ty1 RNA pseudoknots interacting via two 7-base-pair intermolecular RNA duplexes formed between apical motifs in stem-loop SL1a (pink arc) and SL3a (orange arc). The pseudoknot stems are shaded in blue.

The location of hairpin SL3a within an essential packaging domain prompted us to look for features that could function in the formation of a Ty1 RNA kissing complex. We noticed that the ACAGAAU (293/299) sequence in the SL3a loop is perfectly complementary to an AUUCUGU (19/25) motif in the loop and two apical base-pairs of the SL1a stem (G-U and U-A). The tertiary structure of the pseudoknot might allow these complementary motifs to pair intramolecularly. However, 4 of the 7 nucleotides (296/299) in the SL3a loop are highly reactive in SHAPE analysis of RNA in vitro (Figure 2) [20]; therefore, it is unlikely that the SL1a and SL3a motifs are base-paired in vitro. The loop of SL3a is also highly reactive in virio [21], suggesting that the SL1a and SL3a motifs are also not base-paired in VLPs. Another intriguing possibility is that the complementary apical motifs of SL1a and SL3a base-pair intermolecularly to form a symmetrical Ty1 RNA kissing complex with two kissing loops (Figure 3). In vitro, where the TIPIRT domain RNA is monomeric in the absence of Gag [17,40], and in VLPs, where the Ty1 RNA is a mature dimer [10,21], the motif in SL3a is mostly reactive, arguing against base-pairing of the complementary SL1a-SL3a motifs in these RNA forms. Nonetheless, pairing between the SL1a and SL3a apical motifs on different Ty1 RNA molecules could form a transient symmetrical kissing complex that initiates packaging of Ty1 RNA into VLPs, and then is converted to a stable dimer linkage within the mature VLP.

### 3.2. Conservation of Ty1 RNA TIPIRT Domain

**Figure 4:**
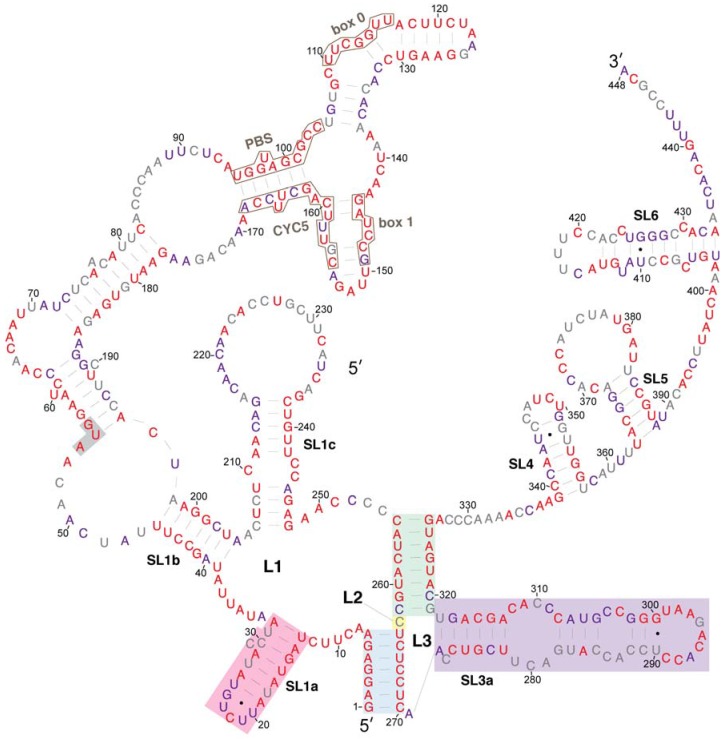
Relative evolutionary conservation of each nucleotide overlayed on the secondary structure model of the 5′ leader of Ty1 RNA. The color of each RNA base indicates its degree among conservation among 102 Ty1 and Ty2 elements from the genus *Saccharomyces.* Categories of conservation are as follows: red, 100% conserved among 102 Ty1 and Ty2 elements in the genus *Saccharomyces*; purple, 100% conserved in 66 *Saccharomyces cerevisiae* Ty1 elements; grey, not 100% conserved in either set.

We compared the conservation of nucleotides within the Ty1 RNA 5′ terminus to the secondary structure model to ascertain whether there are conserved structural features that could function in *cis* in retrotransposition. Because most *S. cerevisiae* Ty1 elements are mobile or recently mobile [41], and therefore have a high degree of sequence identity [42,43], we also compared their sequences to that of Ty2 elements, a closely related family of LTR-retrotransposons in *S. cerevisiae*. The 5′ terminal sequence of 66 Ty1 elements and 32 Ty2 elements from a variety of laboratory, industrial and natural *S. cerevisiae* strain genomes [44], as well as four Ty1 elements from other *Saccharomyces* species were aligned. Each nucleotide position was assigned to one of three categories based on the degree of conservation at that position: (1) conserved in all 102 *Saccharomyces* Ty1 and Ty2 elements (Figure 4, red coloring); (2) conserved in all 66 *S. cerevisiae* Ty1 elements (Figure 4, purple coloring); or (3) variable among the *S. cerevisiae* Ty1 elements analyzed (Figure 4, grey coloring). 

The alignment indicates that nucleotides in the pseudoknot core are very highly conserved. S1 nucleotides are invariant in all *Saccharomyces* Ty1 and Ty2 elements. S2 nucleotides, including C255 and G326, whose pairing is predicted uniquely in the structural model presented here, are invariant, with the exception of three nucleotides at the base of S2. Two of these nucleotides (C262 and C320) are substituted in a few Ty2 elements, while the third nucleotide, G319, is a U nucleotide in four of the 66 *S. cerevisiae* Ty1 elements, but is otherwise conserved. Similarly, the L2 nucleotide C263 is substituted by an A nucleotide in three *S. cerevisiae* Ty1 elements. Thus, every residue of the pseudoknot core is invariant or has limited variation, in agreement with the conclusion of Huang et al. [20].

The entire 326-nucleotide pseudoknot domain has a high degree of conservation overall. Sequences that are very highly conserved among *S. cerevisiae* Ty1 elements include those that bind tRNA_i_^Met^ (PBS, Box 0 and Box 1; Figure 4, black outlines) and those within sequence regions that are predicted to be base-paired, including the SL1a stem, regions of the SL1b stem such as the pairing between nucleotides 39–45 and 198–204 and the SL1c stem. While most regions that are predicted to be single stranded have low nucleotide conservation, nucleotides 8–12, nucleotides 34–38, nucleotides 63–69, and the SL3a loop are conserved. The SL1a loop is conserved in *S. cerevisiae* Ty1 elements but not in Ty2 elements. Within the 53-nucleotide 5′ UTR, 34 nucleotides (64%) are invariant amongst all 102 *Saccharomyces* Ty1 and Ty2 elements analyzed, while 44 nucleotides (83%) are conserved among 66 *S. cerevisiae* Ty1 elements.

### 3.3. Requirement for Pseudoknot Stems S1 and S2 in Retrotransposition

**Figure 5:**
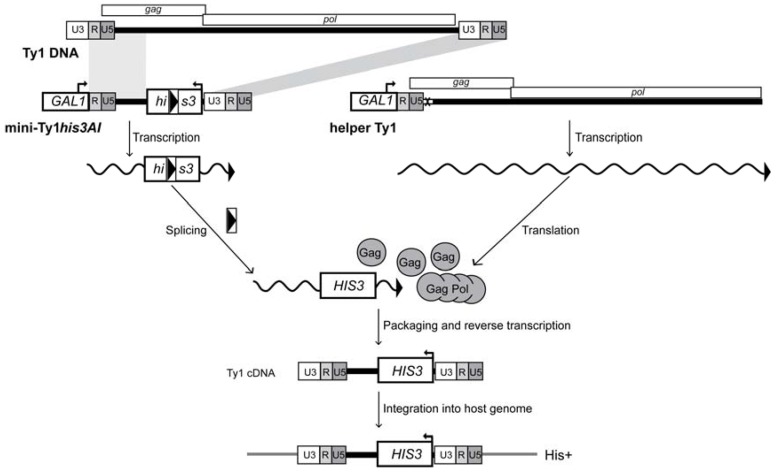
Assay for helper-mediated retrotransposition of mini-Ty1*his3AI*. A complete Ty1 element is shown at the top for reference. The mini-Ty1*his3AI* element and helper-Ty1 element are each expressed from the *GAL1* promoter (labeled rectangle), which is fused to the transcription start site of Ty1-H3 at the first nucleotide of the R domain in the 5′ LTR. *GAL1*:mini-Ty1*his3AI* is carried on a *URA3*-based plasmid and *GAL1*:helper-Ty1 is contained on a *TRP1*-based plasmid (not illustrated). The elements are co-expressed in an *spt3∆* strain lacking endogenous Ty1 element transcription. The internally deleted mini-Ty1*his3AI* element contains 5′ sequences corresponding to nucleotides 1–575 of Ty1 RNA, as well as the last 357 nucleotides of Ty1, including the 3′ polypurine tract (not illustrated) and 3′ LTR. The *his3AI* retrotransposition indicator gene, consisting of the *HIS3* marker gene interrupted by an antisense intron (boxed arrowhead), is inserted in the mini-Ty1 between the 5′ leader and 3′ LTR. The direction of mini-Ty1*his3AI* transcription from the *GAL1* promoter (denoted by the arrow atop the *GAL1* rectangle) is opposite to the direction of *his3AI* transcription (denoted by an arrow atop the *HIS3* rectangle), so the intron is only be spliced from the Ty1*his3AI* transcript. The helper-Ty1 element carries functional *GAG* and *POL* ORFs, but the polypurine tract and 3′ LTR are deleted. In addition, silent nucleotide substitutions in the PBS (denoted by a white rectangle marked with an “X”) block the binding of tRNA_i_^Met^. Splicing is illustrated by removal of the boxed arrowhead representing the intron from the rectangle that denotes the *HIS3* gene. Gag and Gag-Pol proteins translated from the helper-Ty1 RNA form VLPs that package the spliced mini-Ty1*HIS3* RNA, which is reverse transcribed to form Ty1*HIS3* cDNA. Integration of the cDNA into the host genome allows the cell to be detected as a His^+^ prototroph.

To identify the role of Ty1 RNA secondary structures in retrotransposition, we used an established helper-Ty1/mini-Ty1 assay in which two defective but complementing Ty1 elements are co-expressed, each from a plasmid-based *GAL1* promoter (Figure 5) [11]. The helper-Ty1 element encodes functional Gag and Gag-Pol proteins, and its RNA is packaged in VLPs but cannot be used in reverse transcription because it harbors silent substitutions in the PBS and lacks the 3′ polypurine tract and LTR [11]. The mini-Ty1*his3AI* element has an internal deletion of most of the *GAG* ORF and the entire *POL* ORF; nonetheless, 5′ leader sequences corresponding to nucleotides 1–575 of Ty1 RNA as well as the last 357 nucleotides of Ty1, including the 3′ polypurine tract and LTR, are retained. Together, these regions are sufficient for mini-Ty1 RNA to be used as a template for retrotransposition when Ty1 proteins are supplied in trans. Mini-Ty1*his3AI* also carries the *his3AI* retrotransposition indicator gene, which allows cells harboring transposed reverse transcripts to be detected as His^+^ prototrophs [45]. The plasmids were expressed in an *spt3∆* strain, which lacks expression of endogenous Ty1 RNA. The median retrotransposition frequency in the strain co-expressing the mini-Ty1*his3AI* with wild-type sequences and the helper-Ty1 was 1.82 × 10^−6^. The frequency of His^+^ prototrophs in the absence of helper-Ty1 was 1.8% of that in its presence. This background of His^+^ prototrophs may be due to a low frequency of recombination events that introduces full-length genomic Ty1 sequences into the mini-Ty1*his3AI* plasmid.

**Figure 6:**
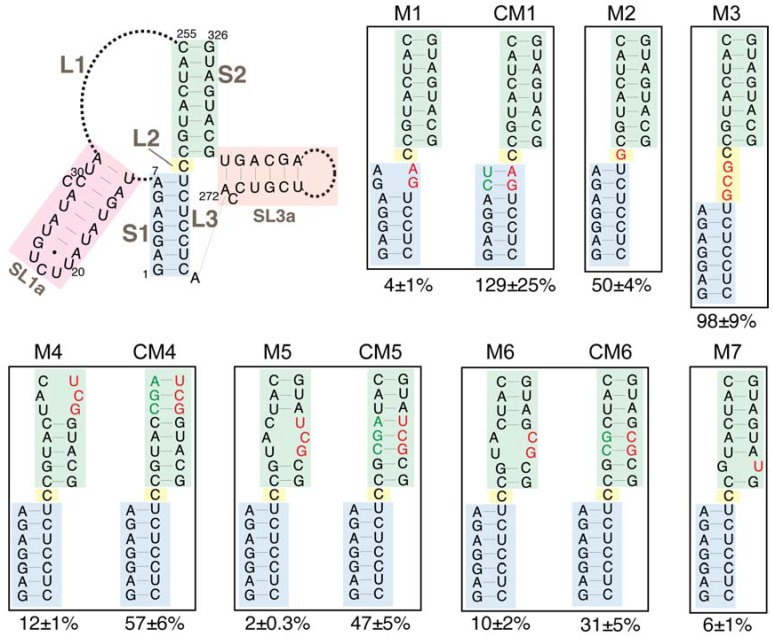
Retrotransposition of mini-Ty1*his3AI* elements with mutations in the Ty1 pseudoknot core. The schematic (top left) shows the secondary structure of the Ty1 pseudoknot core and portions of the L1 and L3 loops. Blue shading, stem S1; yellow shading, loop L2; green shading, stem S2; pink shading, SL1a hairpin, a segment of the L1 loop; orange shading, SL3a hairpin, a portion of the L3 loop. Dotted lines represent bases in loops L1 and L3 that are not shown. Labeled, boxed schematics show the nucleotide substitutions or additions in each mutant mini-Ty1*his3AI* element analyzed. Black letters represent wild-type nucleotides; red letters represent nucleotide substitutions or additions; and green letters represent compensatory substitutions that restore base-pairing with nucleotide substitutions. The percentage below each box is the median frequency of helper-mediated retrotransposition of the mini-Ty1*his3AI* bearing the indicated mutation divided by the median helper-mediated retrotransposition frequency of the mini-Ty1*his3AI* element with wild-type Ty1-H3 sequence, +/− the 95% confidence interval.

Mutations were introduced into structural elements of the TIPIRT domain of the mini-Ty1*his3AI* plasmid. All mutations and compensatory mutations introduced into *GAG* maintained an open reading frame but not necessarily the amino acid sequence of the truncated Gag product. An UC264AG substitution that disrupts S1 complementarity in mini-Ty1*his3AI* RNA reduced helper-Ty1 mediated retrotransposition to 4% of that of the mini-Ty1*his3AI* with wild-type sequence (Figure 6, M1). A compensatory mutation that reestablishes S1 complementarity restored retrotransposition to levels equivalent to the wild-type mini-Ty1*his3AI* (Figure 6, CM1). Similar results were obtained with the identical substitutions in a previous study [20]; therefore, these findings validate the helper-Ty1/mini-Ty1 assay and confirm the role of the S1 pairing in retrotransposition [11,19].

We analyzed the requirement for pseudoknot stem S2 by introducing double and triple nucleotide substitutions that disrupt S2 complementarity. These mutations reduced retrotransposition to 2–12% of that of the wild-type mini-Ty1*his3AI* (Figure 6, M4, M5 and M6). Even the single C320U substitution, which is predicted to change a G-C base-pair to a G-U base-pair, reduced retrotransposition to 6% of wild-type activity (Figure 6, M7). Reestablishing S2 complementarity in the mutants harboring double and triple nucleotide substitutions by introduction of compensatory mutations restored retrotransposition up to 31–57% of the wild-type mini-Ty1*his3AI* (Figure 6, CM4, CM5 and CM6). Compensatory mutations may not fully reconstitute the activity of the wild-type mini-Ty1*his3AI* because the base composition of S2 or ensemble folding of mini-Ty1*his3AI* RNA is altered. Together, these data suggest that the S2 stem of the pseudoknot is as critical for retrotransposition as the S1 stem.

Many pseudoknots have 0 to 1-nucleotide interhelical loops that promote a stable pseudoknot conformation in which individual stems stack coaxially [46]. It has been suggested that S1 and S2 of the TIPIRT domain pseudoknot stack coaxially [20,21], even though the unreactive L2 nucleotide can be substituted without major effects on pseudoknot structure or function [20]. To determine the consequences of disrupting the potential for coaxial stacking of the pseudoknot stems, we increased the length of L2 from one to four nucleotides by addition of a GCG triplet (Figure 6, M3). This mutation had no effect on retrotransposition of mini-Ty1*his3AI*. We also confirmed that the C236G substitution of the L2 nucleotide reduced retrotransposition only modestly (50%) (Figure 6, M2). In summary, our data demonstrate that neither the length nor composition of L2 is a major determinant of pseudoknot conformation; therefore, coaxial stacking of S1 and S2 is not likely to be necessary for pseudoknot function.

### 3.4. Requirement for Complementary Motifs in SL1a and SL3a Hairpins in Retrotransposition

**Figure 7:**
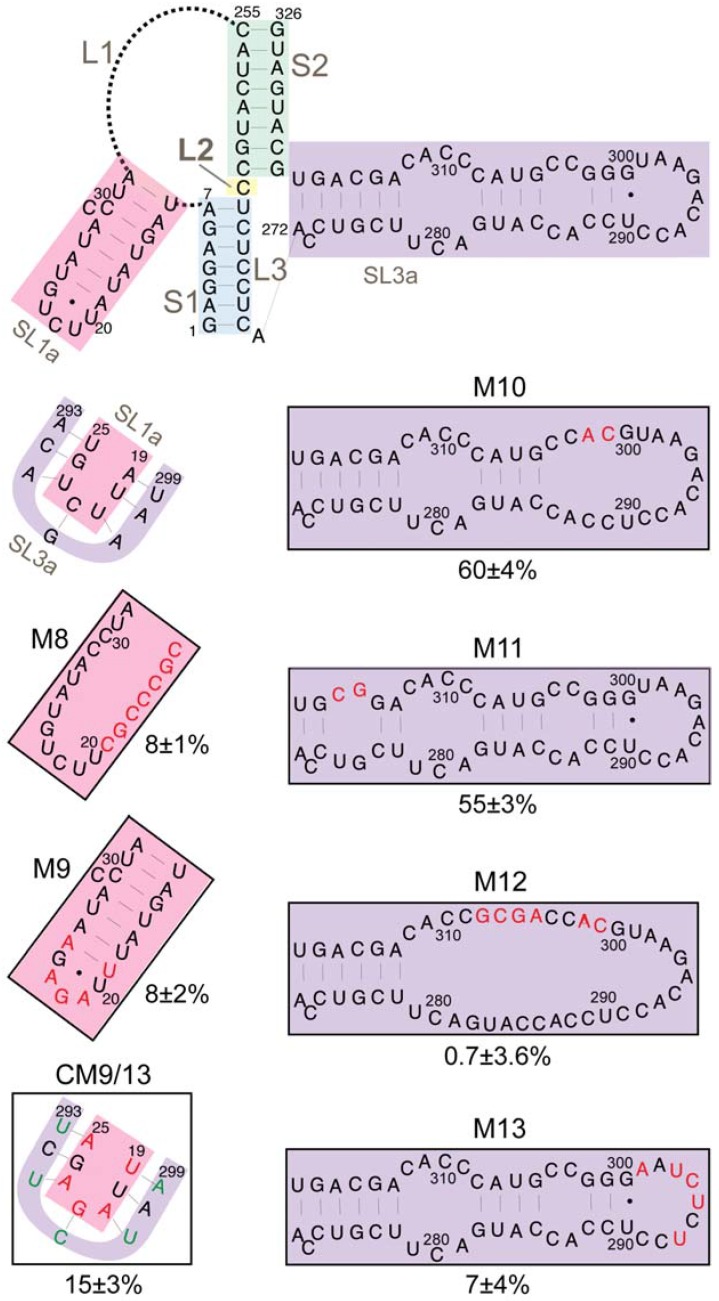
Retrotransposition of mini-Ty1*his3AI* elements with mutations in stem-loops SL1a and SL3a. The schematic (top) shows the secondary structure of the Ty1 pseudoknot core and loops L1, L2 and L3, with the SL1a hairpin (pink shading) and SL3a hairpin (purple shading) highlighted. A second schematic (second from top, left) shows the proposed kissing loop interaction between the seven apical nucleotides of hairpin SL1a (pink shading) and seven apical sequences of the SL3a hairpin. Labeled, boxed schematics show the nucleotide substitutions or additions in each mutant mini-Ty1*his3AI* element analyzed. Black letters indicate wild-type sequence; red letters indicated nucleotide substitutions or additions; and green letters indicate compensatory substitutions that are predicted to restore base-pairing with nucleotide substitutions. The percentage below each box is the median frequency of helper-mediated retrotransposition of each mini-Ty1*his3AI* bearing the indicated mutation divided by the median helper-mediated retrotransposition frequency of the mini-Ty1*his3AI* element with wild-type Ty1-H3 sequence, +/− the 95% confidence interval.

The SL3a hairpin (272/318) is in a region of the TIPIRT domain that contains essential Ty1 RNA packaging sequences [11]. The ACAGAAU (293/299) motif in the loop of SL3a is complementary to the AUUCUGU motif (19/25) encompassing the 3-nucleotide loop and first two base-pairs of the SL1a stem (Figure 7). Except for 1 nucleotide (G296) in SL3a, both sequences are invariant in *S. cerevisiae* Ty1 elements. Therefore, we hypothesized that intermolecular “kissing loop” interactions between the complementary sequences in SL1a and SL3a (Figure 3) could initiate dimerization of Ty1 RNA. To determine whether these complementary motifs are individually required for retrotransposition, we substituted UCUCUAA for ACAGAAU (293/299) in the SL3a loop, which reduced helper-Ty1-mediated mini-Ty1*his3AI* retrotransposition to 7% of wild-type activity (Figure 7, M13). Substitution of UUAGAGA for AUUCUGU (19/25) in SL1a reduced retrotransposition to 8% (Figure 7, M9). Both the AUUCUGU19UUAGAGA mutant and wild-type RNA have an A-U and G-U base-pair at the apex of the SL1a stem; thus, the retrotransposition defect of the AUUCUGU19UUAGAGA mutant is probably not due to disruption of the SL1a stem. Instead our findings indicate that complementary motifs in SL1a and SL3a are required in *cis* in Ty1 retrotransposition.

To determine whether reestablishing complementarity between apical sequences of the SL1a and SL3a hairpins restores retrotransposition, both AUUCUGU19UUAGAGA and ACAGAAU293UCUCUAA were introduced into a single mini-Ty1*his3AI* element. This double mutant transposed at 15% of the frequency of the wild-type mini-Ty1*his3AI* and about 2-fold more often than either single mutant (Figure 7, CM9/13). Partial restoration of retrotransposition rather than an additive decrease in retrotransposition in the SL1a-SL3a double mutant suggests that base-pairing between complementary apical sequences of SL1a and SL3a promotes retrotransposition. Restoration of retrotransposition is not as strong as that seen with other compensatory mutations in stem S1 or S2 of the pseudoknot, but such a difference is expected if the SL1a-SL3a interaction is intermolecular, as opposed to the intramolecular interactions that form stem S1 and S2. This is because a mini-Ty1 RNA bearing both SL1a and SL3a mutations would only be able to form a kissing complex with another mutant mini-Ty1 RNA and not with the wild-type helper-Ty1 RNA, and therefore the pool of kissing complexes that could be packaged into VLPs would be reduced. However, these data alone cannot differentiate between an intramolecular or intermolecular interaction between of the SL3a loop and complementary sequences in the SL1a stem-loop.

To examine the role of the SL3a bulged stem in retrotransposition, we introduced double mutations near the base and the loop of the SL3a stem. Nucleotides C324 and A325, and the bases with which they are predicted to pair (275/276) are invariant among Ty1 and Ty2 elements; however, disruption of this pairing caused only a minor decrease in retrotransposition (Figure 7, M11). Similarly, a two-nucleotide substitution of CA for GG (301/302) near the SL3a loop also resulted in a minor retrotransposition defect (Figure 7, M10). In contrast, substitution of six nucleotides within the bulged stem of SL3a strongly decreased retrotransposition (Figure 7, M12).

Sequences that comprise the SL1a stem-loop are mostly conserved, particularly in *S. cerevisiae* Ty1 elements, despite the fact that this region is non-coding. A 7-nucleotide substitution that completely disrupts pairing in the S1 stem strongly reduced retrotransposition (Figure 7, M8). Mini-Ty1*his3AI* RNA with a two-nucleotide substitution in the SL1a stem could not be co-transformed with helper-Ty1 into the same yeast strain, even though several transformation strategies were attempted. In summary, major nucleotide substitutions in the stems of SL1a and SL3a hairpins strongly decreased retrotransposition, but it remains to be determined whether the secondary structure of the stems is the critical feature required.

### 3.5. Role for the S2 Stem and SL1a-SL3a Kissing Loops in Ty1 RNA Stability

**Figure 8:**
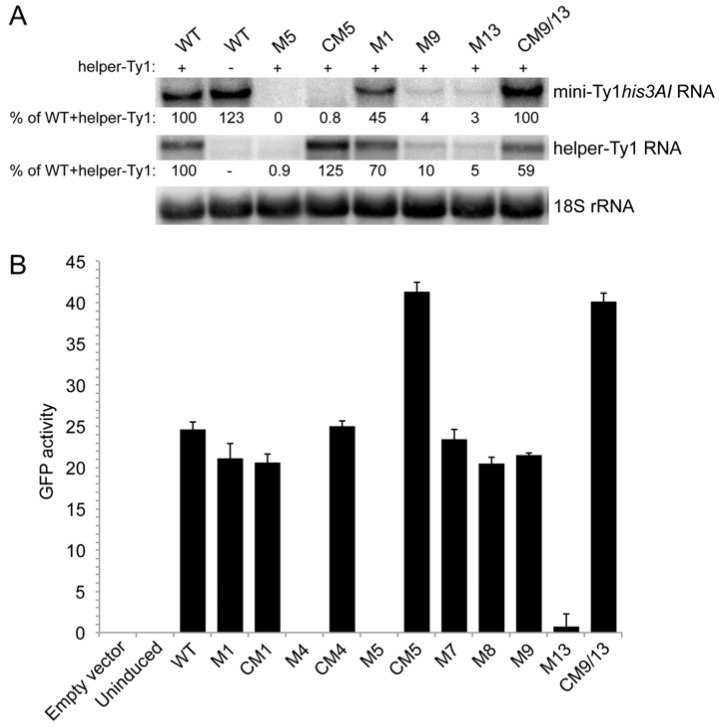
Levels of mini-Ty1*his3AI* RNA bearing different mutations and helper-Ty1 RNA. (**A**) Northern blot analysis of strains carrying the p*GAL1*:mini-Ty1*his3AI* plasmid harboring wild-type Ty1 sequences (WT) or mutant Ty1 sequences and the p*GAL1*:helper-Ty1 induced for 24 hours in galactose-containing medium. The presence or absence of the p*GAL1*:helper-Ty1 plasmid is indicated by + and – symbols, respectively, above the blot. Labels for mutations correspond to those in Figure 6 and Figure 7; (**B**) Measurement of the median GFP activity in 10,000 cells of two different transformants of each p*GAL1:GAG_NT_:GFP* plasmid containing wild type Ty1 TIPIRT domain sequences or derivatives with mutations named as in Figure 6 and Figure 7. Strains were induced in galactose-containing medium for 2.5 h. Error bars are the standard deviation of the median GFP activity in each of two transformants.

Because the S2 stem and SL3a hairpin overlap with a region required for Ty1 RNA packaging, mutations in the S2 stem and SL3a loop, as well as apical mutations in the SL1a hairpin hypothesized to interact with SL3a, might inhibit retrotransposition by blocking packaging of Ty1 RNA. To explore this possibility, we first determined whether mutations in stem S2 and hairpins SL1a and SL3a affect RNA stability. The level of transcript from wild-type and mutant p*GAL1*:mini-Ty1*his3AI* elements was monitored by northern analysis using a probe specific to *his3AI*. Helper-Ty1 RNA was also quantitated using a probe in the Ty1 *POL* region; a discrete band of ~5.5 kb was detected despite the absence of the termination signal in the 3′ LTR. Strains were induced by growth in galactose for 24 h at 20 °C to mimic the conditions used in the retrotransposition assay. Levels of mini-Ty1*his3AI* RNA in the presence and absence of helper-Ty1 RNA were equivalent (Figure 8A, compare WT lanes plus (+) and minus (−) helper-Ty1), demonstrating that packaging of mini-Ty1*his3AI* RNA is not required for stability. The level of mini-Ty1*his3AI* RNA with a UC264AG mutation in pseudoknot stem S1 was decreased about 2-fold (Figure 8A, M1). This result is consistent with previous analyses of this and other stem S1 mutations in a full-length p*GAL1*:Ty1*his3AI* element in the absence of helper-Ty1 [20]. Thus, disruption of stem S1 minimally affects Ty1 RNA stability. In contrast, mini-Ty1*his3AI* RNA bearing the AUG321GCU mutation in stem S2 was undetectable (Figure 8A, M5). Surprisingly, helper-Ty1 RNA was also absent, indicating that expressing mini-Ty1*his3AI* RNA with the AUG321GCU mutation destabilizes helper-Ty1 RNA in *trans*. Mini-Ty1*his3AI* RNA with double compensatory mutations AUG321GCU/CAU258AGC was also present at very low levels, but the level of helper-Ty1 RNA in this strain was completely restored (Figure 8A, CM5). These findings support the idea that base-pairing of stem S2 is necessary for mini-Ty1 RNA and helper-Ty1 RNA stability. Instability of the AUG321GCU/CAU258AGC mini-Ty1 RNA was unexpected, because this compensatory mutant transposes at 47% of the frequency of the wild-type mini-Ty1*his3AI*. A possible explanation for this inconsistency is that two temporally or structurally distinct pools of the AUG321GCU/CAU258AGC mutant exist, one that is successfully packaged into VLPs and is used in retrotransposition, and another that is degraded.

To explore this possibility, we used a second, more sensitive approach to measure mini-Ty1 RNA levels, this time in the absence of helper-Ty1. The Ty1 sequences from each p*GAL1*:mini-Ty1*his3AI* plasmid was subcloned into an expression plasmid, creating an in-frame fusion of the 5′ UTR and first 522 nucleotides of *GAG* to the *GFP* ORF (Gag_NT_:GFP). The p*GAL1*:mini-Ty1(Gag_NT_:GFP) plasmids were introduced into the *spt3∆* strain, and expression was induced for 2.5 h in galactose at 20 °C. The mean GFP activity in 10,000 cells bearing a plasmid with wild-type or mutant Ty1 sequences was measured by flow cytometry to monitor the presence of Ty1 RNA after a brief galactose-induction (Figure 8B). The GFP activities in isolates with plasmid p*GAL1*:mini-Ty1(Gag_NT_:GFP) containing the UC264AG mutation or the UC264AG/GA6UC compensatory mutation in stem S1 were comparable to that of the plasmid with wild-type Ty1 sequence (Figure 8B, compare M1 and CM1 to WT), supporting the idea that mutations in pseudoknot stem S1 minimally destabilize Ty1 RNA [20]. A single nucleotide substitution at the base of stem S2, which changes a GC pair to a GU pair and decreases retrotransposition to 6% of wild-type also had no significant effect on GFP levels (Figure 8B, M7). However, two triple mutations that disrupt pseudoknot stem S2, AUG321GCU and AUG324GCU, yielded GFP activities that were not detectable above the background fluorescence in a strain without *GFP* (Figure 8B, compare M4 and M5 to empty vector). These results mirror those seen for the AUG321GCU mutant (M5) in northern analysis and imply that disrupting stem S2 substantially destabilizes Ty1 RNA. In contrast, Gag_NT_:GFP levels were restored to 100% or more of wild-type levels in strains carrying the double compensatory mutants, AUG321GCU/CAU258AGC or AUG324GCU/CAU255AGC in stem S2 (Figure 8B, CM4 and CM5). The AUG324GCU/CAU255AGC mutant RNA may be unstable when assayed by northern analysis (Figure 8A, CM5), but able to express Gag_NT_:GFP because of a temporal lag between synthesis and degradation of the RNA, which is sufficient to allow AUG324GCU/CAU255AGC mutant RNA to be packaged and used for retrotransposition. Alternatively, it is possible that co-expression of helper-Ty1 is necessary for instability of the AUG321GCU/CAU258AGC mutant. Overall, these data suggest that disruption of the S2 stem results in rapid degradation of the mini-Ty1 RNA and promotes degradation of helper-Ty1 RNA in *trans*.

Northern blot analysis also revealed that levels of the mini-Ty1*his3AI* RNA and the helper-Ty1 RNA were reduced ten-fold or more in mutants carrying the AUUCUGU19UUAGAGA substitutions at the apex of hairpin SL1a or the ACAGAAU293UCUCUAA substitutions in the SL3a loop of mini-Ty1 RNA (Figure 8A, M9 and M13). Moreover, levels of both the mini-Ty1*his3AI* and helper-Ty1 RNA were rescued in the compensatory mutant with restored SL1a/SL3a complementarity (Figure 8A, CM9/13). The ACAGAAU293UCUCUAA substitutions in SL3a also resulted in very low GFP activity in the Gag_NT_:GFP assay; however, the AUUCUGU19UUAGAGA mutation in SL1a resulted in nearly wild-type levels of GFP activity (Figure 8B, M9 and M13). Interestingly, a 7-nucleotide substitution that disrupts the stem of hairpin SL1a also yielded Gag_NT_:GFP activity that was similar to that of the wild-type plasmid (Figure 8B, M8). The Gag_NT_:GFP activity of the AUUCUGU19UUAGAGA/ACAGAAU293UCUCUAA compensatory mutant is also similar to that of wild-type, suggesting that instability of the ACAGAAU293UCUCUAA mutation in SL3a is rescued by the compensatory mutation in SL1a (Figure 8B, CM9/13). Together, these findings suggest that the apices of SL1a and SL3a hairpins interact via 7 nucleotides of complementarity, and that lack of complementarity destabilizes Ty1 RNA in *cis* and in *trans*. Comparison of the northern and GFP assay results suggest that RNA with mutations in SL1a may be degraded more slowly than those in the SL3a loop or only degraded in the presence of the helper-Ty1. Overall, these data suggest that the S2 stem and kissing loop interactions between SL1a and SL3a may promote an intermolecular interaction between Ty1 RNAs, and that a symmetrical kissing complex with two SL1a-SL3a duplexes may be optimal for Ty1 RNA stability, particularly in the presence of Gag protein.

## 4. Discussion

This study reveals the conservation of sequence motifs and structural elements within the long-range pseudoknot in the TIPIRT domain of Ty1 RNA and describes novel functions for elements within the pseudoknot. We show that the pseudoknot stems can be separated by four nucleotides with no effect on retrotransposition and that mutations that disrupt pseudoknot stem S2 give rise to RNA instability phenotypes that are distinct from phenotypes that result from S1 mutations [19,20]. A major new finding of this work is that mutations that disrupt the S2 stem of the RNA pseudoknot or complementarity between apical sequences of a hairpin in pseudoknot loop L1 (SL1a) and a hairpin that comprises most of pseudoknot loop L3 (SL3a) not only inhibit retrotransposition but also destabilize mini-Ty1 RNA in *cis* and helper-Ty1 RNA in *trans*. Moreover, compensatory mutations that restore pairing in stem S2 or complementarity between SL1a and SL3a apices alleviate Ty1 RNA degradation in *cis* and in *trans* and suppress the retrotransposition defect of single mutants. Based on these findings, we propose a model in which two intermolecular interactions between complementary apical sequences in SL1a and SL3a form a symmetrical kissing complex (Figure 3), and that this kissing complex initiates Ty1 RNA dimerization and packaging. Furthermore, we propose that formation of only a single intermolecular SL1a-SL3a kissing loop targets both interacting RNAs for degradation. This model explains the phenotypes of apical SL1a and SL3a hairpin mutants and mutants with substitutions in the pseudoknot S1 and S2 stems as follows. When the mini-Ty1 with wild-type sequences is expressed, both homogeneous kissing complexes containing two mini-Ty1 or two helper-Ty1 RNAs and heterogenous kissing complexes with one mini-Ty1 RNA and one helper-Ty1 RNA are expected to form, since helper-Ty1 RNA has wild-type SL1a and SL3a sequences and can be packaged into VLPs [11]. We propose that mini-Ty1 mutants with nucleotide substitutions in complementary sequences of either SL1a or SL3a would not be able to form homogeneous mini-Ty1 RNA kissing complexes, and heterogeneous mini-Ty1/helper-Ty1 RNA kissing complexes would have only a single kissing loop, thereby targeting both RNAs for degradation. In the mini-Ty1 RNA with restored complementarity between SL1a and SL3a hairpins, both types of homogeneous kissing complexes could form, but heterogeneous mini-Ty1/helper-Ty1 RNA complexes could not form, even with a single kissing loop, and therefore we propose that neither mini-Ty1 nor helper-Ty1 RNA would be targeted for degradation. The fact that only homogeneous mini-Ty1 RNA kissing complexes would result in retrotransposition events could explain why the compensatory SL1a-SL3a mutant retrotransposes at a much lower frequency than the wild-type mini-Ty1, which can form both homogeneous mini-Ty1 RNA and heterogeneous mini-Ty1/helper-Ty1 RNA kissing complexes that lead to retrotransposition. In the case of the S2 stem, nucleotide substitutions that disrupt base-pairing may block formation of the SL3a stem-loop, as SL3a encompasses all but one nucleotide of the L3 loop between S1 and S2. Indeed, the SL3a hairpin is not present in a 1482 nt in vitro Ty1 transcript that lacks a pseudoknot [21]. One possible interpretation of these data is that the S2 stem is required for SL3a to form. In contrast, the SL1a stem-loop is predicted to form in the absence of a pseudoknot [21]. Therefore, it is possible that mini-Ty1 RNA with mutations that disrupt S2 do not form homogenous mini-Ty1 kissing complexes but instead form heterogeneous mini-Ty1/helper-Ty1 RNA complexes with one kissing loop, targeting both RNAs for degradation. Compensatory mutations that restore complementarity in the S2 stem would allow both SL1a and SL3a hairpins to form, allowing both heterogeneous and homogeneous complexes with two kissing loops to form. Although steady-state levels of RNA from a compensatory mutant in S2 are low, the RNA is stable long enough to express wild-type GFP levels in the Gag_NT_:GFP assay, and, more importantly, the corresponding element is transpositionally active, indicating that at least some mini-Ty1 RNA survives packaging and functions as a template for retrotransposition. Finally, mutations in the S1 stem would not cause degradation of mini-Ty1 RNA despite the fact that the pseudoknot cannot form because neither the SL1a hairpin nor the SL3a hairpin depends on S1 stem formation [21]. Thus, S1 stem mutants could interact heterogeneously and homogeneously with two kissing loops, but retrotransposition would be blocked by a failure of reverse transcription to occur [20,21].

Notably, the complementary 7-nucleotide motifs in SL1a and SL3a are completely conserved within *S. cerevisiae* Ty1 except for one nucleotide (G296) in the SL3a loop; however, nucleotides 19–25 in SL1a are not conserved in Ty2 elements. Thus, if our model for the initial dimerization of Ty1 RNA is correct, the divergence between Ty1 and Ty2 RNA sequences in SL1a and SL3a could impede the packaging of Ty1 and Ty2 RNAs together in the same VLP where template switching during reverse transcription could create chimeric elements. Therefore, failure to form Ty1/Ty2 RNA dimers could explain how these elements are maintained as distinct families. 

It is important to note that the data presented do not include physical evidence that the SL1a and SL3a hairpins interact intermolecularly. Nonetheless, we have shown that substitutions in the SL1a or SL3a apical motifs of mini-Ty1 destabilize helper-Ty1 in *trans*, and importantly, introduction of the corresponding co-varying substitutions in the mini-Ty1 RNA SL3a or SL1a motifs, respectively, complement the RNA instability defect of helper-Ty1 RNA in *trans*. Trans-complementation of the helper-Ty1 defect provides direct genetic evidence of an intermolecular interaction that has not been observed in monomeric Ty1 RNA or in dimeric packaged Ty1 RNA, suggesting that this essential interaction could occur within the transient Ty1 RNA kissing complex. Formally, it is also possible that intramolecular pairing between complementary SL1a and SL3a motifs enhances kissing complex formation, perhaps by promoting an RNA tertiary structure that is necessary for an intermolecular interaction between unidentified regions of the Ty1 TIPIRT domain. Although beyond the scope of this study, many aspects of the model we have proposed might be tested using the in vitro RNA dimerization assay of Cristofari et al. [17], as the RNAs bearing SL1a and SL3a mutations may be stable in vitro.

Retroviral RNAs typically form dimers that are packaged into nascent virions via one or two kissing loop interactions; the resulting kissing complex is converted to a stable dimer during proteolytic maturation of the viral particle [47]. Consistent with retroviral RNAs, Ty1 elements bearing a mutation that blocks proteolytic processing of Gag form dimers, but they are less stable than those formed in wild-type VLPs [10]. These findings suggest that the Ty1 RNA dimer also exists in two forms: an initial kissing complex that is recognized for packaging by the immature Gag protein and a mature dimer that is stabilized during proteolytic maturation of the VLP. Based on these findings, we suggest that two RNA duplexes formed between the complementary 7-nucleotide motifs in SL1a and SL3a result in formation of the initial kissing complex that undergoes a structural transition to the mature form of the Ty1 RNA dimer, which may no longer contain SL1a–SL3a duplexes. Purzyka et al. [25] have proposed that the dimer within VLPs contains interactions between the self-complementary PAL1 and PAL2 sequences within the SL1a stem, as well as a second interaction between PAL3 sequences, which are downstream of the pseudoknot in an area not strictly required for packaging. A possible mechanism that might explain the structural transition between SL1a-SL3a duplexes in the kissing complex and PAL1 and PAL2 duplexes in the mature dimer is that the melting of the first two base-pairs of the SL1a stem by SL1a-SL3a duplex formation could destabilize pairing in the rest of the SL1a stem. Melting of the SL1a stem would expose four of the six PAL1 and PAL2 nucleotides on each strand for duplex formation, and these partial PAL1 and PAL2 duplexes could then be extended by melting the remaining two base-pairs that are interacting with SL3a sequences. 

Is Gag involved in the formation of the Ty1 kissing complex in vivo? Our data suggest that packaging of Ty1 RNA is not required for its stability, since the truncated Gag protein encoded by mini-Ty1 cannot form VLPs [48], yet mini-Ty1 RNA expressed in the absence of Gag from endogenous or helper-Ty1 elements is as stable as in its presence. This conclusion contrasts with that of Checkley et al. [49], who showed that Gag supplied in *trans* enhances the stability of a Ty1 RNA containing a premature stop codon adjacent to the start codon, rendering it untranslatable. It seems likely that our differing conclusions stem from the use of different Ty1 RNAs (mini-Ty1 versus untranslatable Ty1 RNA). In our system, it is possible that mini-Ty1 RNA molecules interact intermolecularly via two SL1a-SL3a duplexes in the absence of Gag, and this could stabilize the RNA. This would explain why mutations in the SL3a loop and S2 stem are unstable in the absence of Gag (Figure 8B). Notably, retroviral dimer initiation sites interact in vitro in the absence of Gag, and it has been argued that kissing interactions of retroviral RNA precede packaging [47,50]. However, dimerization of mini-Ty1 RNA in vitro is not detected in the absence of Gag or a C-terminal fragment of Gag harboring the nucleocapsid domain [17,40]. These findings are consistent with an alternative model in which kissing complex formation in vivo requires Gag binding. In this model, mini-Ty1 RNA would be stable either when kissing complexes do not form in the absence of Gag or when symmetrical kissing complexes form in the presence of Gag, but not when asymmetrical kissing complexes with one SL1a-SL3a duplex form. Notably, many of the mutants analyzed in the pseudoknot core and SL1a stem-loop are in Ty1 RNA sequences that are bound by Gag or the nucleocapsid domain [21,40], suggesting that altered binding of Gag to asymmetrical kissing complexes could be a contributing factor in the degradation of Ty1 RNA in *cis* and in *trans*.

Several lines of evidence confirm the conclusion that the Ty1 pseudoknot forms both in vitro in truncated Ty1 RNA leader sequences and in vivo in mini- and full-length Ty1 RNA and is biologically relevant [20,21]. First, the pseudoknot is predicted by several RNA structure prediction algorithms, even in the absence of constraints imposed by SHAPE reactivities, suggesting it is thermodynamically stable. Second, the core of the pseudoknot is almost completely unreactive, which suggests that both stems of the pseudoknot are base-paired within the same molecule of RNA. Third, both pseudoknot stems are required for efficient retrotransposition of Ty1 RNA in vivo [20]. While the findings suggest that the pseudoknot forms in vivo, they do not rule out the possibility that the individual stems form at different times and act at different steps in retrotransposition. For example, the L1 loop and S1 stem of the pseudoknot include all the 5′ sequences known to be required for initiation of reverse transcription [19,20], while the S2 stem and L3 loop coincide with an essential packaging region [11]. While the SL1a stem within the L1 loop has also been proposed to play a role in packaging, this stem-loop likely forms in the absence of the SL1 stem or pseudoknot [21]. In contrast, stem-loop SL3a does not form in the absence of the pseudoknot [21], and our data clearly suggest that the S2 pairing, like the SL3a kissing motif, is required for Ty1 RNA stability (Figure 8). A role for the individual pseudoknot stems in demarcating and stabilizing two separate structural domains is appealing because of the overlap between structurally and functionally defined domains that has been revealed in this and previous studies [11,19,20,21]. Formation of the pseudoknot versus formation of only the S1 stem or the S2 stem are not mutually exclusive possibilities, and there may be switching between one conformation that is stabilized by the pseudoknot, and others that contain only the S1 stem and the L1 loop, or only the S2 stem and the L3 loop. The idea that formation of the TIPIRT domain pseudoknot is regulated at different points in retrotransposition is attractive because the length and base composition of the 1-nucleotide interhelical L2 loop can be altered without substantial effects on retrotransposition. One interpretation of this finding is that the L2 nucleotide allows for a flexible pseudoknot conformation in vivo, and therefore that the tertiary architecture of the TIPIRT domain could change at different stages in the retrotransposition cycle. The ability of the TIPIRT domain to adopt multiple conformations is likely to be important, given the breadth of functions that the TIPIRT domain plays in retrotransposition.

The secondary structure model of the TIPIRT domain predicts that much of the 53-nucleotide 5′ UTR of Ty1 RNA is sequestered by base-pairing, including the pseudoknot S1 stem. The SHAPE-directed structural model described here as well as earlier models revealed significant secondary structure within the 5′ UTR that is potentially inhibitory to ribosomal scanning, including the base-pairing of nucleotides 1 to 7, stem-loop SL1a, base-pairing of nucleotides 39 to 45, and sequestration of the AUG codon in an helix of seven base-pairs and a 1 × 1 internal loop. Moreover, the 5′ UTR and sequences that base-pair to portions of it are very highly conserved in Ty1 elements, especially in regions with secondary structure. The predicted thermodynamic stability of the Ty1 RNA pseudoknot suggest that its formation results in folding of the 5′ terminus into a compact tertiary structure that would render it inaccessible for translation initiation and perhaps even 5′–3′ degradation. The presence of significant secondary structure is unusual in 5′ UTRs of S. cerevisiae genes [51]. Hence, translation of Ty1 RNA, a requisite step in retrotransposition, is not likely to be favored by formation of the pseudoknot. Regulation of the TIPIRT domain structure may play some role in several peculiarities of Ty1 RNA metabolism and function, including the unusually long half-life [52] and the sensitivity of Ty1 RNA translation to loss of translation initiation factor eIF4G1 and 40S rRNA subunit proteins [53,54,55]. Pseudoknots frequently play regulatory roles in gene expression; thus, regulation of the formation of the TIPIRT domain pseudoknot may be a critical factor governing the partitioning of Ty1 RNA between its different functions in translation, packaging and reverse transcription. 

## Supplementary Materials

The following are available online at www.mdpi.com/1999-4915/9/5/93/s1, Figure S1: Secondary structure of Ty1 RNA 5’ leader by pknotsRG, Figure S2: Secondary structure of Ty1 RNA 5’ leader by IPknot, Table S1: raw SHAPE data in SNRNASM format.
