# Supplementary material for: Structure-Function Model for Kissing Loop Interactions That Initiate Dimerization of Ty1 RNA
Source: Viruses. 2017 Apr 26;9(5):93. doi: 10.3390/v9050093 (PMC5454406; doi:10.3390/v9050093)
Supplement: Supplementary file 1 [file viruses-09-00093-s001.zip › viruses-179003_final_supplementary/viruses-179003_supplementary.docx]

Supplementary Materials: Structure-Function Model for Kissing Loop Interactions that Initiate Dimerization of Ty1 RNA

Eric R. Gamache, Jung H. Doh, Justin Ritz, Alain Laederach, Stanislav Bellaousov, David H. Mathews and M. Joan Curcio


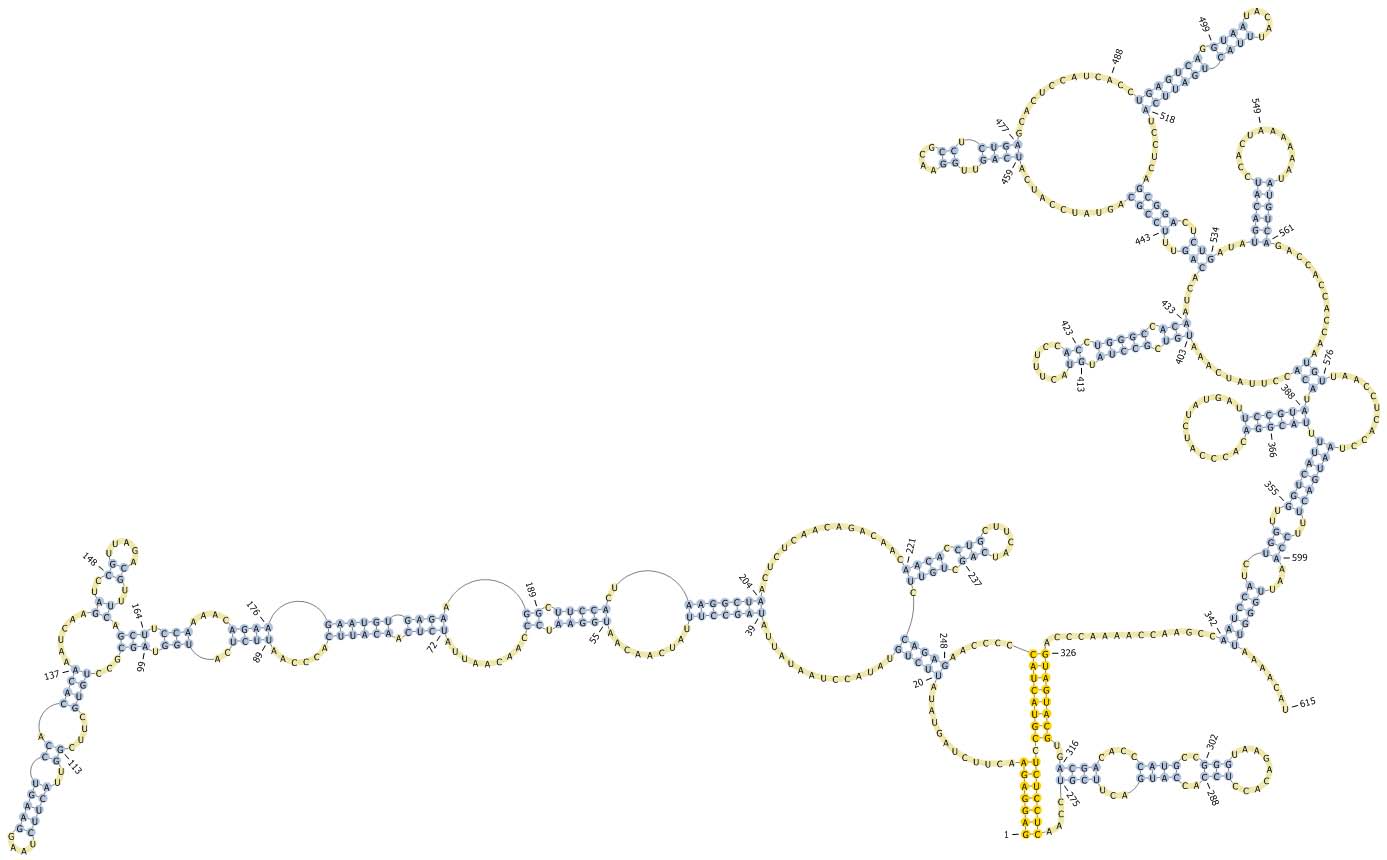


**Figure S1.** Secondary structure of Ty1 RNA 5’ leader by pknotsRG.


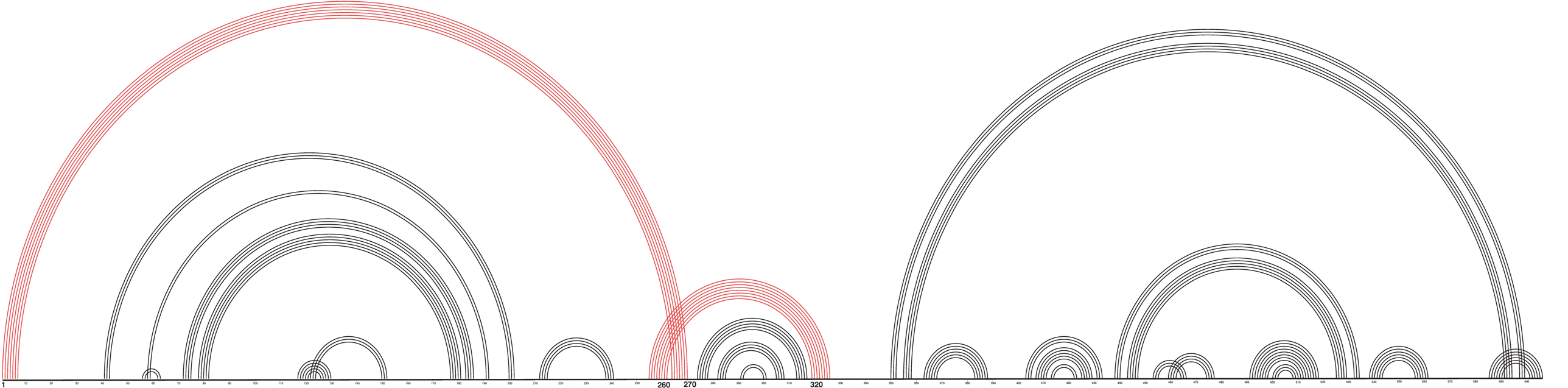

**Figure S2.** Secondary structure of Ty1 RNA 5’ leader by IPknot.

© 2017 by the authors; licensee MDPI, Basel, Switzerland. This article is an open access article distributed under the terms and conditions of the Creative Commons by Attribution (CC-BY) license (http://creativecommons.org/licenses/by/4.0/).
